# No H- and L-type cases in Belgium in cattle diagnosed with bovine spongiform encephalopathy (1999-2008) aging seven years and older

**DOI:** 10.1186/1746-6148-6-26

**Published:** 2010-05-21

**Authors:** Alexandre Dobly, Jan Langeveld, Lucien van Keulen, Caroline Rodeghiero, Stéphanie Durand, Riet Geeroms, Patrick Van Muylem, Jessica De Sloovere, Emmanuel Vanopdenbosch, Stefan Roels

**Affiliations:** 1 Pathology and Prionology, Veterinary and Agrochemical Research Centre, Brussels, Belgium; 2 Central Veterinary Institute of Wageningen UR, Lelystad, The Netherlands

## Abstract

**Background:**

The bovine spongiform encephalopathy (BSE) epidemic presented homogeneity of the phenotype. This classical BSE (called C-type) was probably due to the contamination of the food chain by a single prion strain. However, due to the active surveillance and better techniques, two rare variants of BSE have been recently reported in different continents without a clear correlation to the BSE epidemic. These emerging types behave as different strains of BSE and were named H-type and L-type according to the high and low molecular mass of the unglycosylated fragment of their proteinase K resistant prion protein (PrP^res^). In these types, the proportion of the un-, mono- and di-glycosylated fragments of PrP (glycoprofile) is also atypical and represents an effective diagnostic parameter. This study evaluated the presence of such types in bovine of 7 years and older in Belgium.

**Results:**

The Belgian BSE archive contained 41 bovines of at least 7 years of age. The biochemical features of their PrP^res ^were analyzed by Western blot with five antibodies recognising different regions of PrP^res^, from N- to C-terminus: 12B2, 9A2, Sha31, SAF84 and 94B4. All antibodies clearly detected PrP^res ^except 12B2 antibody, which is specific for N-terminal region 101-105, a PrP region that is only retained in H-types. The glycoprofiles did correspond to that of C-type (with more than 55% of diglycosylated PrP^res ^using antibody 94B4). Therefore, all cases have the features of C-type BSE.

**Conclusions:**

This study supports that, among the BSE cases of 7 years and older identified in Belgium, none was apparently of the H- or L- type. This is consistent with the very rare occurrence of atypical BSE and the restricted dimension of Belgium. These results shed some light on the worldwide prevalence of atypical BSE.

## Background

Prion diseases are infectious neurodegenerative diseases with slow development and lethal outcome. The active agents in this disease family are called prions. Prion diseases are unique as a normal host cellular protein, prion protein (PrP^c^), is usually affected by conformational change and aggregation which leads to the accumulation of PrP^d ^(associated to the disease), usually in the nervous system [1]. There is indeed no specific nucleic acid involved [2]. PrP^d ^is partly resistant to digestion by proteases and the resultant product of such digestion (PrP^res^) is used in diagnostic applications as a highly reliable disease marker [3,4]. Usually, the brain shows microscopic symmetrical spongiosis. Prion diseases are therefore also called transmissible spongiform encephalopathies (TSE). These diseases in animals are mainly transmitted by the dietary route and already described for centuries in sheep as scrapie. Other examples of TSE are chronic wasting disease in deer and elk (CWD), Creutzfeldt-Jakob disease in humans (CJD), transmissible mink encephalopathy and bovine spongiform encephalopathy (BSE), also known as mad cow disease. Evidence for transmission of BSE was found in various other mammals including humans, where it causes a form of CJD - variant CJD (vCJD) - which affects especially young persons and exhibits unique molecular and histological features [5-8].

While in scrapie and other TSEs strain variations were found, BSE initially showed strain homogeneity [9-12]. Indeed, incubation time, vacuolar lesion profile and biochemical signature of PrP^d ^were identical for all investigated cases. This uniform aspect of the BSE prions during the epidemic was probably due to the contamination of the food chain by a single strain in United Kingdom [13].

However, due to the extensive active surveillance and new research techniques, rare variants of BSE have been reported since 2003 [14-24] of which most could be classified in two new types of BSE: H- and L-type [20]. The analysis of their features in bioassay showed that these emerging types represent different strains of BSE [25-28]. Two well identified atypical forms of BSE have been described. Western blot studies showed that, in comparison to classical BSE (C-type), they are characterised by a higher or lower molecular mass of the unglycosylated PrP^res ^and were thus named H-type and L-type BSE [20]. L-type is also called bovine amyloid spongiform encephalopathy or BASE. The PrP^res ^glycoprofile is a very practical marker for the L-type, which shows a much lower proportion of diglycosylated PrP^res ^(<50% of total PrP^res^) than C-type (>55% of total PrP^res^). For H-type, a relatively high reactivity with antibody 12B2 is archetypal because of the characteristic presence of its epitope at the N-terminal end of PrP^res^. A second characteristic of H-type involves a dualistic glycoprofile depending on the antibody used. As the brain area of the PrP^res ^deposition can differ drastically between the L- and C-type [16,29] and potentially also between H- and C-type, it would be better if the sampling techniques presently used are adapted to these atypical types.

Such types are very rare; only 42 cases were described worldwide and these were all detected in old bovines (at least eight years in age) [19-22,30], except one case from a 23 month old cow that could not be further investigated in depth due to shortage of sample [14]. They were reported on different continents. Indeed they might correspond to natural "sporadic" forms of BSE, not originating from the contamination of the food chain [31]. In the past, a 60 month old unclassified BSE case was described in Belgium when molecular typing techniques and comparison with L- and H-types was not available [17]. This sample was re-examined with the current techniques in the present study. It is essential for the surveillance programmes to determine the prevalence on a global level of these atypical types. Therefore this study retrospectively analysed the bovines of at least 7-years-old in the Belgian archive of BSE-diagnosed cattle, using the latter criteria and techniques, in order to determine whether these types of atypical BSE were present in old BSE-positive bovines in Belgium.

## Results

**Figure 1:**
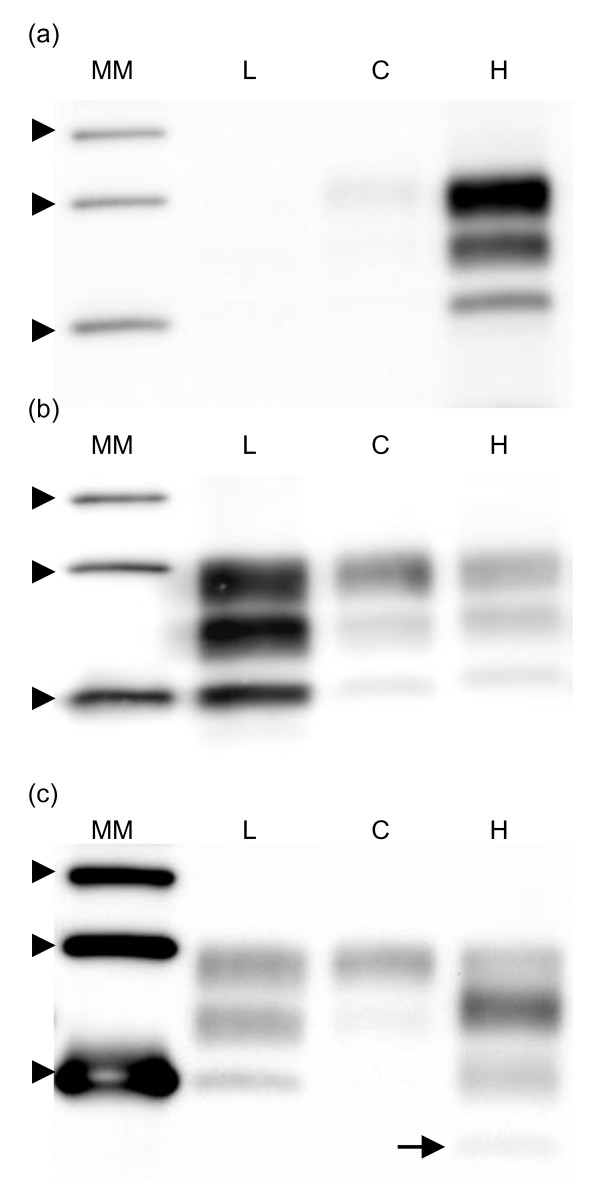
**Western blots comparing C-, L- and H-types**. Western blots comparing reference samples of L-, C- and experimental H-type isolates (indicated by L, C and H). From top to bottom, the three bands of each sample correspond to di-, mono- and unglycosylated forms of the PrP. The different reaction of C-type in comparison to H-type is visible with 12B2 antibody (a), the higher molecular mass of H-type is revealed by Sha31 antibody (b) and the presence of a fourth band in H-type is shown by SAF84 antibody (c, arrow). L-type shows a greater proportion of monoglycosylated band compared to the diglycosylated band with Sha31 antibody than C-type. A molecular marker kit is displayed (MM, three arrow heads represent position of M_r _20, 30 and 40 kDa). The antibody concentrations used were 2 μg/ml for 12B2 and SAF84. For Sha31, the manufacturer's instructions were followed. The applied tissue equivalents are 7.1 mg of fresh brain per lane. The exposure time in the imager was 5 min.

Firstly the H-, L- and C-type reference samples were investigated. In the H-type sample, the unglycosylated band migrated at a higher gel position than in the C-type; moreover the H-type reacted strongly to 12B2 contrary to C- and L-type. It showed a dualistic glycoprofile between group B antibody Sha31 and group C antibody SAF84 (Fig. 1). Furthermore, H-type BSE showed a fourth band of low molecular mass when antibody SAF84 was applied (Fig. 1, arrow). In addition of the absence of 12B2 reactivity as mentioned above, the analysed L-type showed similar glycoprofile with group B and C antibody, with a high proportion of monoglycosylated band (four replicates with Sha31: 38.9%, SD = 3.6 vs. 17.3%, SD = 4.9 for C-type, Fig. 1).

**Figure 2:**
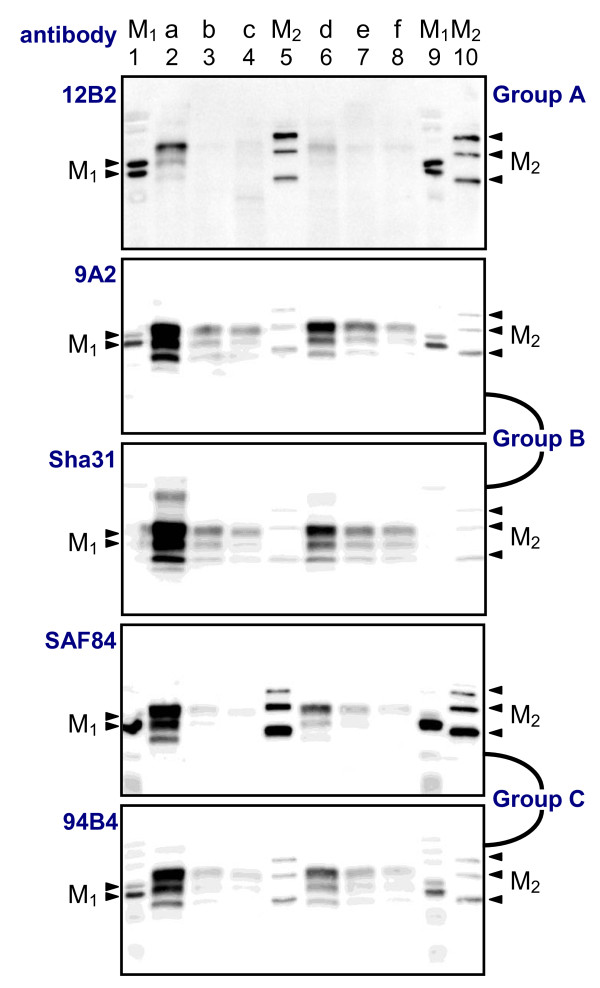
**Differences between the five antibodies used**. Blots showing six bovine C-type BSE samples (a to f) analysed by five antibodies. Two molecular marker kits are each applied twice (lanes 1, 5, 9, and 10; arrow heads indicate for M_1_: positions of 20 and 25 kDa; and for M_2_: 20, 30 and 40 kDa). The groups to which the antibodies belong are shown at the right and have been defined in [30]. Group A antibodies recognise a region of the protein close to the N-terminus, group B recognises the core of the protein while group C antibodies bind in the C-terminal region of PrP. Antibody concentrations were 2, 0.5, 2 and 0.5 μg/ml for 12B2, 9A2, SAF84 and 94B4 respectively, and for Sha31 the manufacturer's instructions were followed. SDS-PAGE gels were 15%, except for Sha31, which was 13.5%. Tissue equivalents applied were 7.1 mg of fresh brain per lane. The strongly positive BSE cases (e.g. samples a and d) can present an additional light grey band below the unglycosylated PrP^res ^band. This corresponds to an unidentified PrP fragment.

The analyses of the Belgian BSE archive did not yield any atypical case. As an illustration, blots obtained with the five different antibodies for seven given samples are presented in Fig. 2. In this figure, PrP^res ^was clearly detected with group B and C antibodies. As it is typical for C-type and L-type BSE, the 12B2 N-terminal epitope is largely removed by proteinase K in all analyzed samples and this is most evident when comparing the signals of 12B2 with those of Sha31.

**Figure 3:**
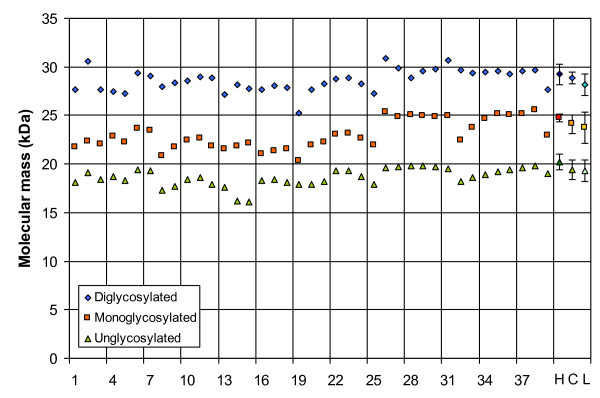
**Apparent molecular masses of the analysed PrP^res^**. Average apparent molecular masses PrP^res ^in the 39 Belgian BSE cases as tested with Sha31 antibody. The values of the three forms are displayed (un-, mono- and diglycosylated forms). The values for H-type (H, darker symbols), C-type (C, normal symbols) and L-type (L, lighter symbols) are based on own results (average and standard deviation of three replicates for each).

**Figure 4:**
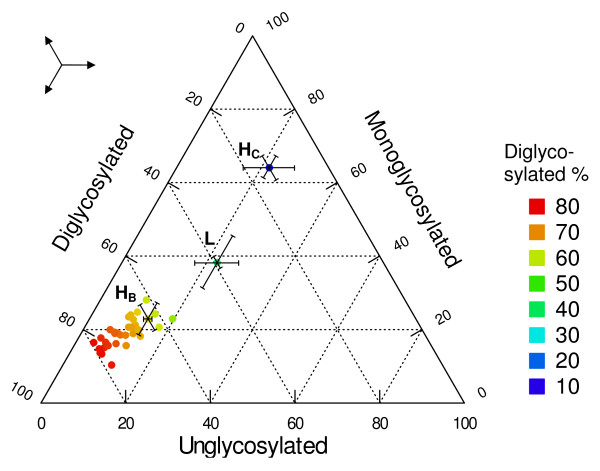
**Glycoprofiles of the analysed PrP^res^**. Glycoprofiles of the 39 resistant prion proteins (PrP^res^), obtained with antibody 94B4. The PrP possesses two, one or no carbohydrates attached. These three forms are found in various proportions in a given animal. Colours refer to the percentage of the diglycosylated form. The average glycoprofiles (with standard deviations) obtained with 94B4 for a reference isolate of H-type and L-type are indicated (H_C _and L, showing lower diglycosylated percentage, <50% for L-type, 3 replicates). H_B _represents the H-type glycoprofile using a group B antibody (Sha31), not allowing a clear discrimination. The three-arrow symbol is to assist the reader in finding the direction of each point to the different axes.

The apparent molecular mass (M_r_) obtained for the 39 samples with Sha31 can be found in Fig. 3. The observed molecular masses display a great variability which can be ascribed to the inaccurate capacity of SDS-PAGE for a reliable estimation of the small M_r _differences between the unglycosylated PrP^res ^bands of different samples even if measured in triplicate or more. As main evidence for discrimination, the group A antibody 12B2 hardly bound compared to group B antibody Sha31, and the glycoprofiles of all the samples exhibited a typical C-type glycoprofile with group B and group C antibodies with >55% diglycosylated PrP^res ^band. To further exemplify this, the glycoprofiles of the individual samples as probed with antibody 94B4 have been plotted in Fig. 4 and compared with the L-type and H-type results. In this last figure, it is clear that the glycoprofiles of the samples did correspond to that of C-type, as characterized by a fraction of diglycosylated PrP^res ^of 55% or more using group C antibody (94B4); the L-type and H-type showed diglycosylated fractions below 50%. Therefore, all cases of age 7 years and older have the features of C-type BSE. The discrimination is easier than in a previous report [20]; this can be due to a better resolution of the blots and thus a more accurate estimation linked to the use of an imager in the present study instead of photographic film plus subsequent digitalisation with a scanner. While another group C antibody (SAF 84) also allowed a straightforward discrimination, a group B antibody (Sha31) did not (see both H-type profiles in Fig. 4). Thus, depending on the antibody used, the H-type sample exhibited a different glycoprofile (dualistic glycoprofile).

A previously reported 5-year old case demonstrated a fast migration of the unglycosylated band at the time [17]. However, reinvestigation of available little left-over material with the current improved tools and knowledge lead to the conclusion that this case does belong to the C-type category (see additional file 1: Blots of published sample).

## Discussion

This study supports that, among the BSE cases of 7 years and older identified in Belgium, none was apparently of the H- or L- type. Unfortunately, three aged BSE cases have not been investigated due to insufficient availability of positive tissue. Nevertheless, as atypical BSE remains very rare, our results are not incongruous. Our previously reported case from [17] reinvestigated with the current improved tools lead to the conclusion that this case does belong to the C-type category.

The results shed some light on the worldwide prevalence of atypical BSE. The absence of atypical cases can be linked to the limited sample size due to the dimension of Belgium, and is also likely to occur when comparing the variable prevalences found in other countries like France (8 L-types, 8 H-types), Poland (6 L-types, 1 H-type), Italy (3 L-types), The Netherlands (2 L-types, 1 H-type), Germany (1 L-type, 1 H-type), United Kingdom (2 H-types), Switzerland (1 H-type) and Catalunya, Spain (none) [20,32]. In all these countries, all cattle of 7 years and older have been tested post-mortem for the presence of BSE using sensitive screening methods. An extensive study already estimated the frequency of H-type and L-type BSE in France to 1.9 and 1.7 per million cattle more than eight years of age [31]. This corresponds to 0.41 and 0.35 atypical cases per million of tested cattle. With almost three million cattle tested in Belgium since 1997, one could have expected to find roughly one H-type and one L-type (i.e. 1 atypical case every five years). It is also possible though difficult to prove, that atypical cases have been missed during the routine surveillance programme due to 1) an unusual location of the PrP^res ^deposition in the brain since at least L-types have a preferential distribution of PrP^res ^in the forebrain region [16] and active surveillance methods are based on the brainstem; and 2) a susceptibility of critical epitopes of PrP^res ^in L- and/or H-type cases to proteinase treatment used for detection combined with the method (TeSeE ELISA of Bio-Rad) used for initial screening (which is dependant on precious protection of an N-terminally located epitope for capturing PrP^res^). With respect to the latter point, screening tests have been developed using C-type cases which appear to be more resistant to digestion with proteinase K than H- and L-type cases [20]. However, it must be mentioned that a significant proportion of L- and H-type cases were detected using this type of capture ELISA [20], the most frequently test used up till 2007. This issue of suitability of screening tests needs nevertheless more attention in future studies when sufficient material is available e.g. from experimentally infected animals.

The age of tested cohort is a determining factor. For example, the cattle population in Poland is older than in other European countries and this country presents high atypical-BSE prevalence (especially of L-types) [21]. However this does not fully explain the prevalence of atypical BSE, as The Netherlands have a higher prevalence with an age structure of bovine population similar to that of Belgium. For now, the importance of the spontaneous aspect of (atypical) BSE remains an open question.

The frequencies of atypical BSE cases are similar to those of the human sporadic Creutzfeldt-Jakob disease (sCJD). Indeed atypical cases were detected in BSE-exposed countries (France, Italy, Germany, The Netherlands, etc. [20]) as well as in a low BSE-exposed country (Sweden [22]). This reinforces the hypothesis of a sporadic origin of atypical BSE [30]. These atypical BSE cases might have existed previously and by their very low frequency remained undetectable for veterinarians, until the introduction of the active surveillance programmes and improvements of diagnostic tools

The origin of the current BSE epidemic could be linked to atypical BSE, especially in view of the properties of L-type (or BASE) [26,27]. It is however also not excluded that the real source of the epidemic was derived from C-type case (which might also have a spontaneous origin), and thus that such cases have always existed sporadically. In such situation when continuing surveillance, C-type cases will be detected in older animals at a stable sporadic level. Potentially brain stem might not be the first site for PrP^res ^development.

About the typing technique used, it is important to note that the apparent molecular mass differences can be useful but present as rather imprecise and impractical criteria for molecular typing of PrP^res ^(Fig. 3). The H-types can be discriminated with that measure if compared with C-types (Fig. 1a). However, given the precision of the Western blot technique, the differences in apparent molecular mass of PrP^res ^for L-types are too small to be undoubtedly detected (0.3 and 0.8 kDa) [18,33]. Other properties yield a more robust approach. For example, the affinity to PrP^res ^to the antibodies from different groups makes H-types easily detectable. Group A antibodies like 12B2 are in comparison to group B antibodies highly reactive due to the N-terminal epitope present, and group C antibodies like SAF84 and 94B4 recognize a fourth band (positioned at about 10-12 kDa). This band corresponds to the unglycosylated moiety of a second population of PrP^res ^triplet. This feature is unique for H-type and thus a fundamental characteristic of this strain [20]. It is not known if this phenomenon occurs in vivo or is a by-product of proteinase K treatment. A powerful discrimination tool remains the comparison of glycoprofiles, i.e. the proportions of the three forms of the PrP. L-types and C-types differ by their proportion of diglycosyl-PrP^res ^which is respectively at/below and above 50% of total PrP^res^. These proportions remain visible whether detection is performed with group B or C antibodies. However, H-types have a dualistic glycoprofile with high proportion of diglycosyl moiety when tested with group A and B antibodies and high proportion of monoglycosyl-PrP when tested with group C antibodies (Fig. 4). In H-types the dualistic phenomenon is linked to the presence of a second PrP population with a very short C-terminal region detected only by C-terminus specific antibodies. The three PrP^res ^moieties of this second population (PrP^res^-2) are 5-10 kDa smaller in molecular mass than those of the first population (PrP^res^-1) and are only detectable by group C antibodies like SAF84 and 94B4. Consequently the migration position of diglycosylated band in PrP^res^-2 coincides with that of the monoglycosylated band of population PrP^res^-1 [20,30].

The emergence of atypical types of BSE is due to the active surveillance screening, a better awareness of prion strain variations, and more efficient diagnostic techniques. However considering molecular properties, PrP^res ^in atypical cases is more susceptible to proteinase K treatment and the area in the brain where PrP^res ^is deposited differs at least between C- and L-types. It is therefore essential to ascertain that the routine sampling and analytical techniques are adapted to these new types. As these new strains seem more virulent than classical types, at least in mice models [27,34-36], they represent one of the next challenges in the field of prions.

## Conclusions

This analysis of the old bovines in the Belgian archive did not show any atypical BSE case in these cohorts. The study implied 42 bovines of at least 7 years of age. Even with the restricted size of Belgium, one could have expected a few atypical cases (as observed in The Netherlands). This difference can be random or linked to an unknown particularity of our samples. Anyway, the results help to estimate the worldwide prevalence of atypical BSE.

## Methods

### Animals and tissues

From the Belgian archive of the 133 BSE-positive bovines, the animals of 7 years and older were selected (N = 41, see Table 1). In Belgium, until 2007, all BSE surveillance laboratories used the Biorad TeSeE ELISA kit for BSE case detection. Based on the availability of remnant suitable brain material, 38 bovines could be analysed; they were aged 93 months old in median (min: 84, max: 181 months). They were detected from August 1999 up till October 2006, when the last BSE case in Belgium was found. The median detection date was November 2002. Among the 41 cattle, ten were 8 years old and ten were 9 years or older. We also added to the present analysis the bovine having shown unusual features in a previous analysis [17]. This animal was 64 months old.

**Table 1:** Distribution of the 41 bovines according to their age (in years)

Age	7-7.4	7.5-7.9	8-8.4	8.5-8.9	9-9.4	9.5-9.9
Total	9	13	7	3	3	0

						

Age	10-10.4	10.5-10.9	11-11.4	11.5-11.9	12-12.4	15-15.4

Total	3	1	0	0	2	1

* indicate the three unanalysed samples. Due to absence of cases, five empty cohorts before the last one are not indicated. The 64-months-old bovine analysed in 2004 is not included.

### Tissue analyses

First the approximate PrP^res ^concentration in brain homogenates was estimated by an ELISA test (Bio-Rad: TeSeE, ref 355 1144 and 355 1194) following the manufacturer's instructions. This TeSeE ELISA carried out on the samples confirmed all of them as positive. The median optical density was 14 times higher than the cut-off (minimum: 1.48). Only three samples were slightly close to the cut-off (1.48 to 2.30 times the cut-off).

Then the study was carried out with a Western blot test-kit (Bio-Rad: Bovine WB, ref 355 1169) modified with a panel of PrP specific antibodies. The antibodies used, namely 12B2, 9A2, Sha31, SAF84, 94B4 are known to detect different regions of the PrP. These antibodies are classified in three groups following a previous categorisation [30] from N- to C-terminal PrP epitope specificity: group A (12B2) to region ± 86-108, B (9A2, Sha31) to region ± 108-157 and C (SAF84 and 94B4) to region ± 157-242. The use of these antibodies allows an efficient typing diagnostic.

Briefly, 250 μl of 20% homogenates were incubated with 250 μl of proteinase K solution at 37°C for 10 min. After the addition of 250 ml of reagent B, the samples were centrifuged at 15000 g for 7 min. The pellets were resuspended by incubating them during 5 min in 100 μl of Laemmli loading buffer [37]. The pellet was dissolved by pipetting and scratching with the tip. Then the samples were heated at 100°C for 5 min. After vortexing, the samples were centrifuged at 15000 g for 15 min. Then 15 μl of the supernatants, heated at 100°C, were run on a 13.5 or 15% sodium dodecyl sulphate polyacrylamide gel according to Laemmli [37] (Fig. 1). The proteins were then transferred on nitrocellulose membranes. The membranes were blocked by 5% non-fat dry milk (1 h, for 12B2 and SAF84), by 3% serum albumine bovine (1 h, for 9A2 and 94B4) or by kit blocking solution (30 min, for Sha31). The membranes were then incubated with the antibodies for 30 min. After three washes in PBS-Tween, the membranes were incubated for 20 min for most antibodies with a mixture of horseradish-peroxidase-labelled conjugate (for antibody detection: goat anti-mouse Ig(H+L), Clinisciences 1010-05, Montrouge, France) and streptavidin-peroxidase (for molecular mass marker detection). Sha31 was detected using the kit conjugate. After four washes in PBS-Tween and one in PBS, the bound antibodies were detected with chemoluminescence (SuperSignal West Dura, Pierce) in Versadoc (Bio-Rad). The PrP^res ^signals were analysed with an image-analysis software (Quantity One, Bio-Rad). The apparent molecular mass was determined using two molecular mass marker kits (Magic Mark, Invitrogen and Precision Plus Protein Kaleidoscope, Bio-Rad). The tissue equivalent applied per lane was 7.1 mg of fresh brain.

As a primary validation, the method used in this study was controlled by comparing known C-types to an experimental H-type generated at CVI-WUR, Lelystad, The Netherlands and an L-type diagnosed in Poland [21] (kindly provided by Miroslaw Polak). Our intended analysis was based on the most crucial characteristics for a correct typing of PrP^res^: firstly the apparent molecular mass of the un-, mono- and diglycosylated bands (higher in H-types, lower in L-types) using group B antibody, secondly the binding behaviour of the antibodies for PrP^res ^compared to that of antibody Sha31 (e.g. 12B2 reacts only well with PrP^res ^of H-types, SAF84 detects a fourth band in H-types), and thirdly the glycoprofile based on the relative proportions of the visible bands (a lower diglycosylated proportion is characteristic for L- and H- type BSE with group C antibodies used, compared to C-type BSE samples), and fourthly, only H-type samples shows two glycoprofiles depending on the antibody used (group A and B antibodies show a C-type glycoprofile, while group C antibodies have a unique glycoprofile); this two-glycoprofile character of H-types was defined as a dualistic glycoprofile behaviour [20]. Alternatively said, a sample is considered as H-type if it shows three clear bands with 12B2 antibody and a fourth band with SAF84 antibody at 10-12 kDa. For L-types, the decisive discrimination criterion was the absence of significant reactivity with group A antibody (12B2) and a glycoprofile of <50% of diglycosylated and ≥ 30% monoglycosylated PrP^res ^with use of either group B (9A2 or Sha31) or group C (94B4 or SAF84) antibody.

## Authors' contributions

AD designed the experiments, adapted the protocols, analysed the data and wrote the manuscript. JL helped to adapt the protocols, advised in antibody use and had an important input into the manuscript. LVK carried out the experimental infection with H-type and revised the manuscript. CR carried out the main laboratory manipulations. SD, PVM and JDS carried out the initial laboratory analyses and prepared the samples. RG cared for the archive and data management. EV revised the manuscript. SR supervised the experiments and revised the manuscript. All authors read and approved the final manuscript.

## Supplementary Material

Additional file 1 **Western blots of the sample previously published**. Western blots comparing the sample previously published in [17] with reference samples of L-, C-, and experimental H-type isolates. Click here for file
