# Supplementary material for: No H- and L-type cases in Belgium in cattle diagnosed with bovine spongiform encephalopathy (1999-2008) aging seven years and older
Source: BMC Vet Res. 2010 May 21;6:26. doi: 10.1186/1746-6148-6-26 (PMC2881063; doi:10.1186/1746-6148-6-26)
Supplement: Additional file 1 — Western blots of the sample previously published. Western blots comparing the sample previously published in [17] with reference samples of L-, C-, and experimental H-type isolates. [file 1746-6148-6-26-S1.PDF]

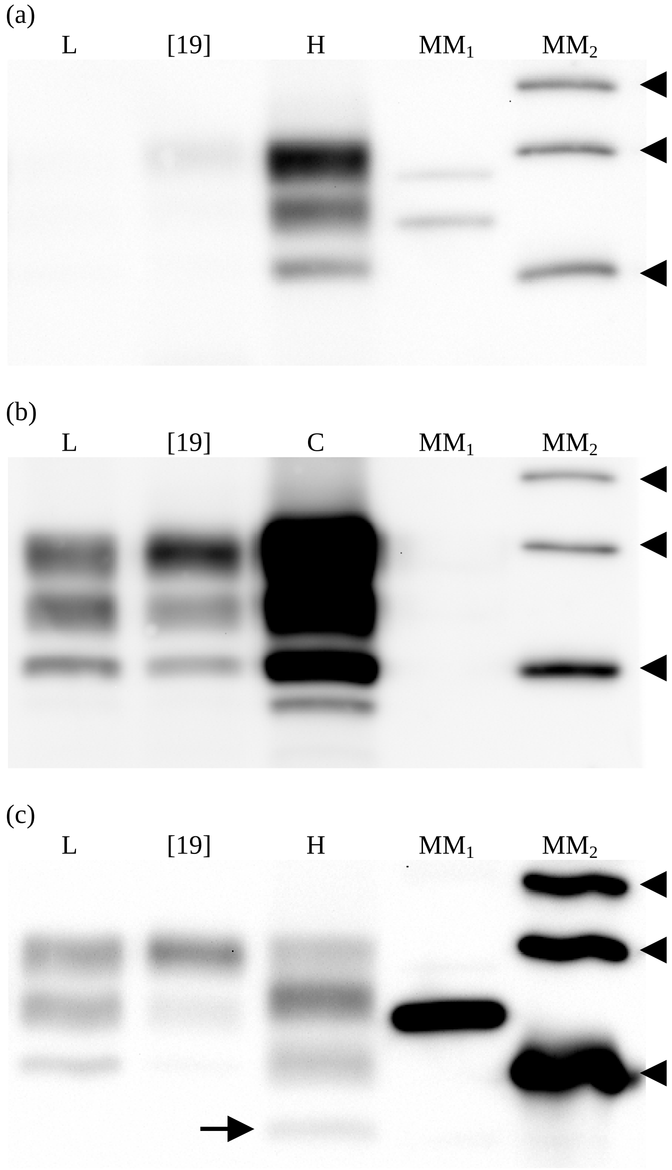

Supplementary figure - Western blots of the sample previously published

Western blots comparing the sample previously published in [17] with reference samples of L-, C- and experimental H-type isolates (indicated by L, C and H). From top to bottom, the three bands of each sample correspond to di-, mono- and unglycosylated forms of the PrP. The different reaction of the sample in comparison to H-type is visible with 12B2 antibody (a), the greater proportion of the diglycosylated band (63.2%, SD = 3.6, n = 3) compared to the monoglycosylated band in the sample, contrary to the L-type, is revealed by Sha31 antibody (b) and the absence of a fourth band in the sample contrary to H-type is shown by SAF84 antibody (c, arrow). The unglycosylated band did not migrate faster than the C-type as previously reported [19]. Two molecular marker kits are also displayed (MM_1 and MM_2, three arrow heads represent position of M_r 20, 30 and 40 kDa in MM_2). The antibody concentrations used were 2 µg/ml for 12B2 and SAF84. For Sha31, the manufacturer's instructions were followed. The applied tissue equivalents are 7.1 mg of fresh brain per lane. The exposure time in the imager was 5 min. In lane C of panel b, the additional light grey band below the PrPres triplet of this C-type case represent an unidentified PrP fragment that is sometimes present in strongly positive BSE cases.
